# Identification of a Mg^2+^-sensitive ORF in the 5′-leader of TRPM7 magnesium channel mRNA

**DOI:** 10.1093/nar/gku951

**Published:** 2014-10-17

**Authors:** Inna A. Nikonorova, Nikolay V. Kornakov, Sergey E. Dmitriev, Konstantin S. Vassilenko, Alexey G. Ryazanov

**Affiliations:** 1 Department of Cellular and Molecular Pharmacology, Rutgers Robert Wood Johnson Medical School, Piscataway, NJ 08854, USA; 2 Institute of Protein Research, Russian Academy of Sciences, Pushchino, Moscow Region 142290, Russia; 3 Belozersky Institute of Physico-Chemical Biology, Lomonosov Moscow State University, Moscow, 119992, Russia; 4 Engelhardt Institute of Molecular Biology, Russian Academy of Sciences, Moscow, 119992, Russia

## Abstract

TRPM7 is an essential and ubiquitous channel-kinase regulating cellular influx of Mg^2+^. Although TRPM7 mRNA is highly abundant, very small amount of the protein is detected in cells, suggesting post-transcriptional regulation of *trpm7* gene expression. We found that TRPM7 mRNA 5′-leader contains two evolutionarily conserved upstream open reading frames that act together to drastically inhibit translation of the TRPM7 reading frame at high magnesium levels and ensure its optimal translation at low magnesium levels, when the activity of the channel-kinase is most required. The study provides the first example of magnesium channel synthesis being controlled by Mg^2+^ in higher eukaryotes.

## INTRODUCTION

TRPM7 is a protein present in vertebrates that functions in the regulation of cellular magnesium homeostasis. TRPM7 polypeptide chain contains two functionally different moieties: an alpha-kinase domain and a region of six transmembrane domains that form an ion channel pore upon tetramerization of TRPM7 molecules at the cell surface (1). TRPM7 ion channel exhibits inward currents of divalent cations (2). The currents are regulated by intracellular concentration of Mg^2+^. They are activated by decreased levels of intracellular Mg^2+^ and strongly inhibited by free Mg^2+^ levels of more than 1 mM (3).

TRPM7 is an essential protein. Truncation of *trpm7* gene results in growth and proliferation arrest in lymphocyte cells in G_1_/G_0_ phase of the cell cycle. This blockage in the cell cycle progression can be rescued by supplementing the culturing media with additional 10 mM Mg^2+^ or by concomitant overexpression of various magnesium transporters such as bacterial MgtE or eukaryotic MagT1 (4–8). Moreover, homozygous TRPM7-deficient mice with deletion of the kinase domain exhibit early embryonic lethality, whereas heterozygous mice survive but have altered magnesium homeostasis and develop signs of hypomagnesaemia. Similar to TRPM7-deficient lymphocytes murine embryonic stem cells with the deletion of the kinase domain cannot proliferate at normal magnesium levels, but are able to do so when cultured under conditions of elevated (10 mM) magnesium concentrations (9).

TRPM7 is ubiquitous and evolutionarily conserved among all vertebrates. TRPM7 transcripts are among the most abundant in cells (10,11); however protein levels of TRPM7 are negligible (12) to such an extent as to make it nearly impossible to detect by biochemical techniques without preliminary enrichment through immunoprecipitation or/and overexpression. The abundance of TRPM7 transcripts together with extremely low protein levels suggests post-transcriptional regulation of *trpm7* gene expression.

In this paper we report that TRPM7 mRNA has two evolutionarily conserved upstream open reading frames (uORFs) within the 5′-leader sequence preceding the start codon of TRPM7 coding region and that these uORFs regulate *trpm7* gene expression at the level of translation. Specifically, we found that the uORFs had two major effects: (i) uORF1 inhibited translation of the main coding sequence in general and (ii) uORF2 inhibited translation of the main coding sequence at elevated Mg^2+^ levels, promoting better translation at reduced Mg^2+^ concentration.

## MATERIALS AND METHODS

### Nucleotide and peptide sequence alignments

Nucleotide sequence alignments were done using ClustalOmega and shaded using BoxShade software. Peptide sequences were aligned using Multalin software.

### Generation of the constructs

The firefly luciferase sequence was purchased from Promega (pGL4.50 vector). TRPM7 5′-leader sequence was recovered from IMAGE Mouse cDNA clone Id 2123372 (accession number for 5′ sequence reading is A1891807, Open Biosystems catalog number EMM1002–205949940). TRPM7 3′-UTR was recovered from Mouse cDNA clone Id 42421999 (accession number for sequence reading is BC026440, Open Biosystems catalog number MMM1013–202705585). All three pieces were spliced by assembly PCR in one chimeric product “TRPM7 5′-leader—*Firefly luciferase*—TRPM7 3′-UTR” and named “M7-Luc-M7”. Additionally, “human β-globin 5′-leader—*Firefly luciferase*—TRPM7 5′-UTR” construct was generated and called “β-Luc-M7”. M7-Luc-M7 construct was modified to obtain a series of mutants carrying different changes in the TRPM7 5′-leader region. The chimeric sequences β-Luc-M7, M7-Luc-M7 and its multiple mutants were cloned into pcDNA3.1 vectors under T7 promoter between KpnI and BamHI restriction sites. Preparations of plasmids for *in vitro* transcription were fully sequenced to make sure that there were no unwanted mutations generated during the process of subcloning and propagation.

### Preparation of mRNAs for translation

RNAs were generated using RiboMax Large Scale Kit (Promega), purified by phenol-chloroform extraction and precipitated by addition of sodium acetate and ethanol as described in standard protocols. Purified RNAs were capped using Vaccinia Capping system (NEB) and then polyadenylated with Poly(A) Polymerase Tailing Kit (Epicentre). Purification from non-incorporated nucleotides was performed with RNeasy Cleanup Kit columns (Qiagen). Concentrations of recovered mRNAs were precisely measured using commercially available fluorescence-based dyes (Invitrogen, Q10210).

### *In vitro* translation in HEK293T system

HEK293T extract was prepared as described in (13). Briefly, cells were harvested at logarithmic phase of growth, washed twice with PBS, partially lysed for 1 min with a lysolecithin-containing buffer (20 mM HEPES-KOH pH7.4, 100 mM potassium acetate, 2.2 mM magnesium acetate, 2 mM DTT, 0.1 mg/ml lysolecithin, Sigma L5254). The cells were then pelleted at 10 000 g for 10 s, resuspended in hypotonic buffer at the ratio 1:1/ v:v (20 mM HEPES KOH pH 7.4, 10 mM potassium acetate, 1 mM magnesium acetate, 4 mM DTT, EDTA-free complete protease inhibitor tablet by Roche) and incubated on ice for 10 min, following disruption using a glass Dounce tissue grinder. Cell debris was pelleted by centrifugation at 10 000 g for 10 min; the supernatant represented ready to use lysate for *in vitro* translation. The lysate was then frozen in liquid nitrogen and stored at −70°C in small aliquots. All of the manipulations described above were done in a cold room on ice using ice-cold wash and lysis buffers.

The translation reaction was performed in the volume of 15 μl and contained 50% HEK293T extract, 20 mM HEPES KOH pH 7.4, 1 mM DTT, 0.5 mM spermidine, 8 mM creatine phosphate, 1 mM ATP, 0.2 mM GTP, 0.025 mM each amino acid mixture, 0.1 mM luciferin, 60 mM of added potassium acetate (if not mentioned otherwise), 0.8 units/μl RiboLock (Thermo Scientific), 0.025 pMol/μl mRNA of interest and magnesium acetate added in different concentrations. Reactions were mixed on ice in 384-well plates. The luminescence signal was collected at 30°C using Tecan Infinite M200 Pro with 1 min intervals for a total of 1 h to reconstruct a kinetic curve of the translation reaction.

### Data analysis

Translation of each mRNA was repeated at least three times in a given lysate over a range of added Mg^2+^ concentrations from 0.2 to 1.4 mM in 0.2 mM increments. To compare Mg^2+^ sensitivity of different constructs we used a maximum synthesis rate parameter, calculated as a maximal gain of luciferase activity per minute achieved in the course of translation reaction (measured in arbitrary luminescence units/ min, ALU/min). The values of maximum synthesis rates were plotted against corresponding magnesium concentrations and interpolated with a bell-shaped curve. The magnesium concentration that provided the highest value of the maximum synthesis rate was referred as the magnesium optimum.

### RNA transfections to HEK293 cells

Transfections were performed as described in (14). mRNAs for transfection reactions were prepared in the same way as for *in vitro* translation. RNA coding for Renilla luciferase was produced by *in vitro* transcription directed by pRL-SV40 vector (Promega) linearized by BamHI restriction site and was further capped and polyadenylated as described above. One hour prior to transfection procedure culturing media of HEK293 cells was changed to an experimental one (either with 0 mM, 0.8 mM or 8.0 mM of Mg^2+^). Transfection procedure was performed in the experimental media using Lipofectamine 2000 delivery agent (Invitrogen) according to manufacturing protocol. Specifically, 1.5 μl of Lipofectamine and 0.5 μg of RNA (ratio of FLuc :RLuc = 10:1) were used to transfect 1.5×10^5^ cells in a well of a 24-well plate. After 3 h cells were washed with PBS, lysed with Passive lysis buffer (Promega) and assayed for the presence of Renilla and Firefly luciferases using Dual-Luciferase Reporter Assay System (Promega). Activity of Renilla luciferase was used to normalize the activity of the Firefly luciferase for transfection efficiency.

## RESULTS

### 5′-leader sequence of TRPM7 mRNA contains two evolutionarily conserved uORFs

**Figure 1.**
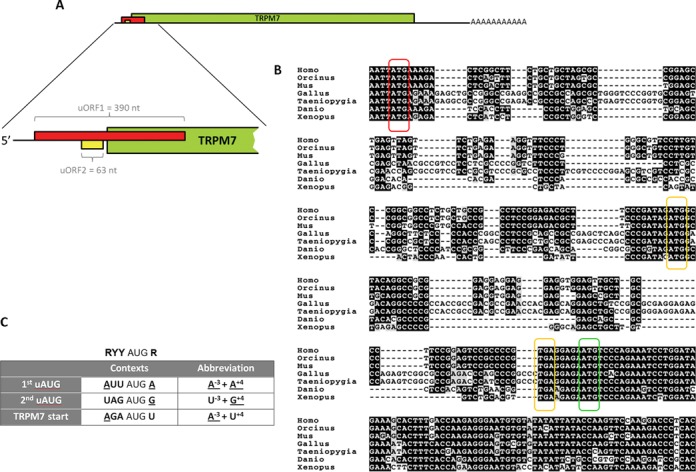
TRPM7 5′-leader contains two uORFs that are highly conserved across all vertebrates. (**A**) Schematic representation of TRPM7 mRNA organization with a close up on 5′-leader sequence. TRPM7 coding sequence is shown in green. The uORF1 (shown in red) overlaps the start codon of TRPM7 coding sequence. The uORF2 (shown in yellow) ends 5 nucleotides before the start codon of TRPM7 coding sequence. (**B**) Alignment of 5′ regions of TRPM7 mRNAs from different vertebrate species. The first uAUG (uAUG1) is marked in red, the second upstream AUG (uAUG2) and the corresponding termination codons are marked in yellow. The main AUG codon of TRPM7 coding sequence is marked in green. (**C**) Highly conserved contexts of the two uAUG codons and the main start codon of TRPM7 mRNA are indicated in the table. Nucleotides that match Kozak consensus for optimal initiation are underlined. Ideal Kozak consensus is presented above the table (R indicates purine nucleotide, Y indicates pyrimidine nucleotide).

TRPM7 mRNA encodes a 220 kD TRPM7 protein with no indications of alternative splicing of TRPM7 pre-mRNA. The coding sequence of TRPM7 protein is more than 5500 bases long and is preceded by a 5′-leader region of over 250 nucleotides. The whole 5′-leader together with the start codon of TRPM7 is contained within the first exon of *trpm7* gene. Analysis of the 5′-leader sequence showed that it possessed some common features of translationally regulated mRNAs: (i) high GC content (67.5%) and (ii) two upstream AUG codons (uAUG). These uAUGs are out of frame with respect to each other and to the TRPM7 protein coding sequence (Figure 1A). Phylogenetic analysis of the TRPM7 5′-leader sequences across vertebrates revealed strong conservation of the uAUG codons and their relative positions (Figure 1B). The first uAUG (uAUG1) codon resides 101 nucleotides from a 5′-end of TRPM7 mRNA and serves as a start codon for a 390 nucleotide long uORF (uORF1). This uORF1 overlaps the main TRPM7 coding sequence by 206 nucleotides (Figure 1A, shown in red). The second uORF (uORF2) starts 117 nucleotides downstream of the uAUG1. It is 63 nucleotides long and ends 5 bases upstream of the TRPM7 start codon (Figure 1A shown in yellow). The exact lengths and positions of the uORFs are given for murine TRPM7 mRNA used in this work.

No conservation was seen in amino acid sequences encoded by the uORF1 and uORF2 of TRPM7 mRNAs across different classes of vertebrates, suggesting that the encoded peptides are unlikely to play an important functional role (Supplementary Figure S1). At the nucleotide level, however, the strongest conservation is seen in the sequences immediately flanking the uAUG codons, suggesting their important roles in the regulation of *trpm7* gene expression. According to classical studies of Kozak (15) the context of the uAUG2 is weakened by the presence of a pyrimidine (uridine) in a critical, −3, position. The uAUG1 is considered to be the strongest among all the three AUG codons of TRPM7 5′-leader as it contains adenine nucleotide in −3 position and a purine nucleotide (also adenine) in another critical, +4, position (Figure 1C), suggesting an inhibitory role for the uORF1.

### TRPM7 5′-leader is inefficient in directing translation of a downstream reporter coding sequence

**Figure 2.**
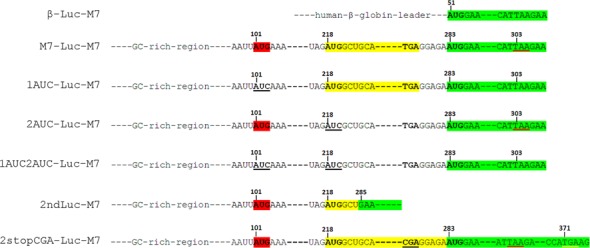
Constructs designed to test the ability of TRPM7 5′-leader to direct translation of a luciferase reporter sequence. The main reading frame, coding for *Firefly* luciferase, is marked in green, the uORF2 is marked in yellow, and the AUG codon of the uORF1 is marked in red. Stop codon that terminates translation of the uORF1 is underlined in red. Mutations of TRPM7 5′-leader are underlined in black. Correspondence of a few key nucleotides of TRPM7 5′-leader mutant forms to positions of the same nucleotides in M7-Luc-M7 construct is indicated with numbers. The random stop codon that terminates translation initiated by the second uAUG in 2stopCGA-Luc-M7 construct is underlined in yellow.

**Figure 3.**
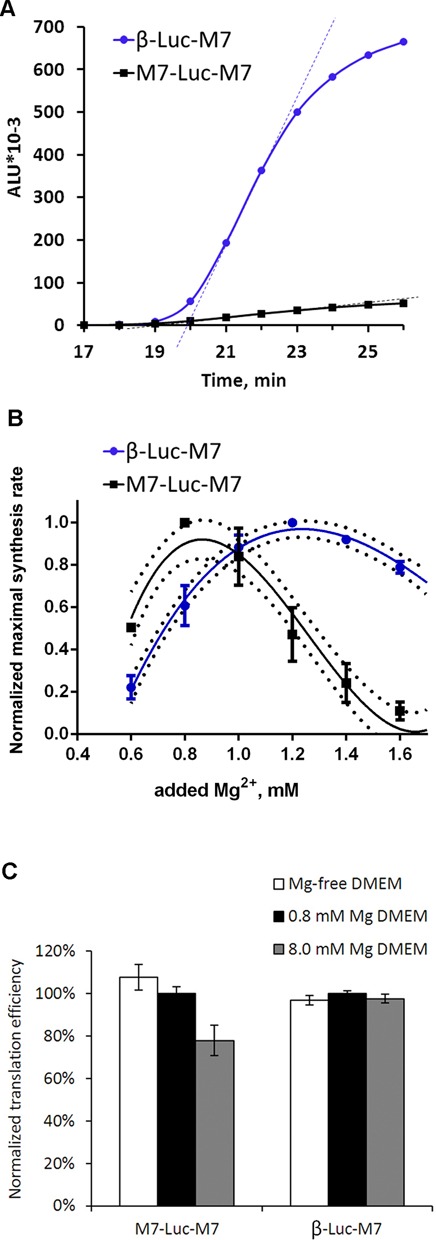
Inefficient translation directed by TRPM7 5′-leader can be activated by lowering Mg^2+^concentration. (**A**) Kinetic curves of translation reactions directed by β-globin (circles) and TRPM7 (squares) 5′-leaders conducted under conditions of 1 mM of added magnesium acetate and 120 mM of added potassium acetate. Dashed lines show the maximal slopes of the kinetic curves, which reflect maximal synthesis rates of translation reactions. (**B**) Dependence of the maximal protein synthesis rates on Mg^2+^ concentration is given for translation reactions carried out at 120 mM of added K^+^. For each construct on diagram B, the maximal synthesis rate at each Mg^2+^ concentration was normalized to the value at the optimal Mg^2+^ concentration. Error bars represent standard deviation (SD) of three experiments. (**C**) Sensitivity of TRPM7 leader-directed translation measured *in vivo* to Mg^2+^ concentration in the medium. Cells were transfected with either β-Luc-M7 or M7-Luc-M7 mRNAs under different culturing conditions (DMEM containing either 0 mM, 0.8 mM or 8.0 mM of magnesium). Columns represent *Firefly* luciferase activity measured 3 h post transfection and normalized to the value of *Renilla* luciferase that was produced from an mRNA cotransfected with the constructs of interest. Error bars represent SD of two independent experiments performed in duplicates.

To investigate the role of the 5′-leader sequence in translation of TRPM7 mRNA, we generated a chimeric construct, which contained a firefly luciferase reporter coding sequence flanked by native 5′-leader and 3′-untranslated region of murine TRPM7 mRNA. The resulting construct M7-Luc-M7 had a shortened uORF1 (203 nt) that overlaps the luciferase coding sequence by 23 nucleotides (Figure 2). To focus on translational control and avoid any contributions from other post-transcriptional events, we employed an *in vitro* translation system prepared from HEK293 cells. In this system we tested the ability of the TRPM7 5′-leader to direct translation of the luciferase coding sequence. Our results showed that the luciferase mRNA with TRPM7 5′-leader was translated far less efficiently than the one with the classical β-globin leader (Figure 3A), confirming the expected inhibitory role of TRPM7 5′-leader in translation of the main coding sequence.

### TRPM7 5′-leader favors translation of the main coding sequence at decreased magnesium concentrations

Since TRPM7 is involved in the maintenance of cellular magnesium homeostasis, we analyzed whether its expression depended on Mg^2+^ concentration. We employed the HEK293 cell-free translation system to measure protein synthesis rate of the TRPM7 leader-directed translation reaction over a range of Mg^2+^ concentrations. Previously it was shown that the maximal synthesis rate parameter reflects the rate of translation initiation and is more reliable than the commonly used estimation of overall protein yield (16). We found that the optimal Mg^2+^ concentration for translation of the mRNA with TRPM7 5′-leader was significantly lower than that for the β-globin leader-directed translation (Figure 3B). These data demonstrate that a decrease in Mg^2+^ levels enhances translation directed by TRPM7 leader. Analogous results were obtained when the same 5′-leaders directed translation of the reporter sequences in cell-free systems prepared from the wheat germ and murine ascite carcinoma Krebs2 cells (Krebs2). In these systems, the TRPM7 leader also inhibited translation of the main coding sequence and promoted its better translation at decreased Mg^2+^ concentrations (Supplementary Figure S2). To test whether such unusual pattern of magnesium dependence is a characteristic of TRPM7 5′-leader, we generated two additional constructs with 5′-leaders of GAPDH and Polr2e cellular mRNAs. Magnesium optimums of translation reactions directed by these leaders were similar to that of translation directed by β-globin leader and higher than the one for translation directed by TRPM7 leader (Supplementary Figure S3).

The observed differences in the efficiency of translation directed by either β-globin or TRPM7 5′-leaders suggest that the latter may represent an intracellular sensor of magnesium concentration and adjust production of TRPM7 according to the cellular needs for this channel. To test whether translation directed by TRPM7 5′-leader is regulated by Mg^2+^ levels *in vivo*, we transfected HEK293 cells with M7-Luc-M7 and β-Luc-M7 mRNAs under conditions of different magnesium concentrations in the culturing medium. Luciferase activity measurement 3 h post transfection procedure revealed inhibitory effect of elevated concentration of magnesium on translation directed by TRPM7 5′-leader, whereas magnesium deficiency, in turn, stimulated translation directed by the leader (Figure 3C). Even though it was shown that varying extracellular magnesium concentration produces limited effect on the global concentration of free intracellular magnesium (17,18), we observed clearly measurable differences in translation efficiency. These differences are likely to come from local or transient changes in intracellular magnesium concentration induced by immediate changes in the culturing media and therefore are not as dramatic as the effects observed in the *in vitro* system (Figure 3B).

### TRPM7 5′-leader as a specific magnesium sensor

**Figure 4.**
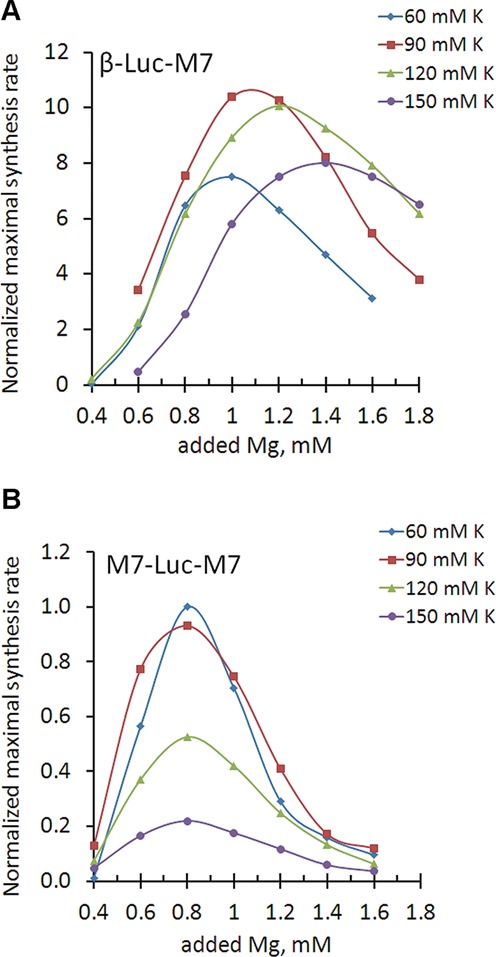
Magnesium optimum of translation directed by TRPM5 5′-leader is not perturbed by varying potassium concentration. Dependence of maximal synthesis rate on magnesium concentration at different potassium levels (diamonds—60 mM, squares—90 mM, triangles—120 mM and circles—150 mM of added potassium acetate). Panel (**A**) represents translation directed by β-globin 5′-leader. Panel (**B**) represents translation directed by TRPM7 5′-leader. Maximal synthesis rates are normalized to the value of M7-Luc-M7 translation at 60 mM of added potassium at the optimal magnesium concentration (0.8 mM of added magnesium acetate). All the reactions used to reconstruct magnesium dependencies on this figure were performed using the same lysate preparation.

As a rule, optimal Mg^2+^concentration in *in vitro* translation systems can be altered by varying monovalent salt levels (19). Thus, we tested whether magnesium optimum of translation directed by TRPM7 5′-leader could be altered by varying concentration of potassium ions. We observed that magnesium optimum of translation of mRNA with β-globin leader was labile as expected, i.e. increasing at higher potassium levels (Figure 4A). Conversely, varying K^+^ concentration did not perturb magnesium optimum of translation directed by the TRPM7 5′-leader (Figure 4B). This observation suggested that TRPM7 5′-leader utilized a specific mechanism to “sense” the surrounding magnesium concentration.

Here we note that we chose to measure maximal synthesis rate dependence on the concentration of added magnesium acetate, not the absolute level of free magnesium in the translation system. The basal concentration of free Mg^2+^ in different cell extracts may not be the same, due to variability in the process of extract preparation. Therefore, each extract was first calibrated by translation of M7-Luc-M7 mRNA to determine the value of added magnesium to promote optimal translation of M7-Luc-M7 in that extract. All other constructs translated in these calibrated extracts were normalized to the value of maximal synthesis rate of M7-Luc-M7 at its magnesium optimum. Our analysis showed that even though the *absolute* value of the added magnesium optimum could vary by ±0.1 mM for each construct when tested using different extracts prepared from the same types of cells, the *relative* magnesium optimums of the different constructs were highly uniform. For example, for all lysate preparations magnesium optimum of β-Luc-M7 at 120 mM of added potassium concentration was always 0.4 mM higher than that of M7-Luc-M7, whereas at 60 mM of added potassium it was always 0.2 mM higher than that of M7-Luc-M7 (Figure 4).

**Figure 5.**
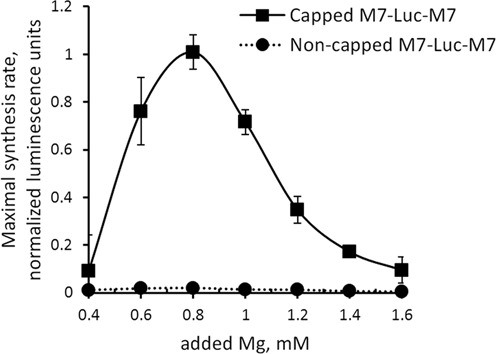
TRPM7 5′-leader utilizes 5′-cap-dependent mechanism of translation initiation. Translation directed by a non-capped construct is inefficient as compared to the capped M7-Luc-M7 construct.

Prokaryotes regulate synthesis of magnesium transporters via riboswitch-mediated mechanisms based on the specific abilities of their mRNA 5′-leaders to adopt alternative conformations under conditions of different magnesium concentrations (20). Although riboswitch-like mechanisms have not been found in eukaryotes, it is possible that TRPM7 5′-leader sequence might also form alternative regulatory structures under conditions of different magnesium levels that would affect translation of the main reading frame, for example by means of internal ribosome entry site (IRES)-mediated mechanisms. Analysis of TRPM7 5′-leader sequence using RNAalifold software (21) predicted no regions with developed secondary structure. Nevertheless, we tested whether translation initiation directed by TRPM7 5′-leader occurs by the classical cap-dependent or an IRES-mediated mechanism. In our previous studies, uncapped transcripts that harbored IRESs in their 5′-UTRs provided as efficient protein synthesis as their capped versions for at least 3 h in HEK293 cells (22). Comparison of the efficiencies of translation reactions programmed with either capped or non-capped M7-Luc-M7 mRNAs demonstrated that TRPM7 5′-leader strictly required the cap structure for efficient translation of the main coding sequence (Figure 5), suggesting that conventional cap-dependent mechanism is utilized to initiate translation.

### Roles of the uORF1 and the uORF2 in the control of translation of the main coding sequence

**Figure 6.**
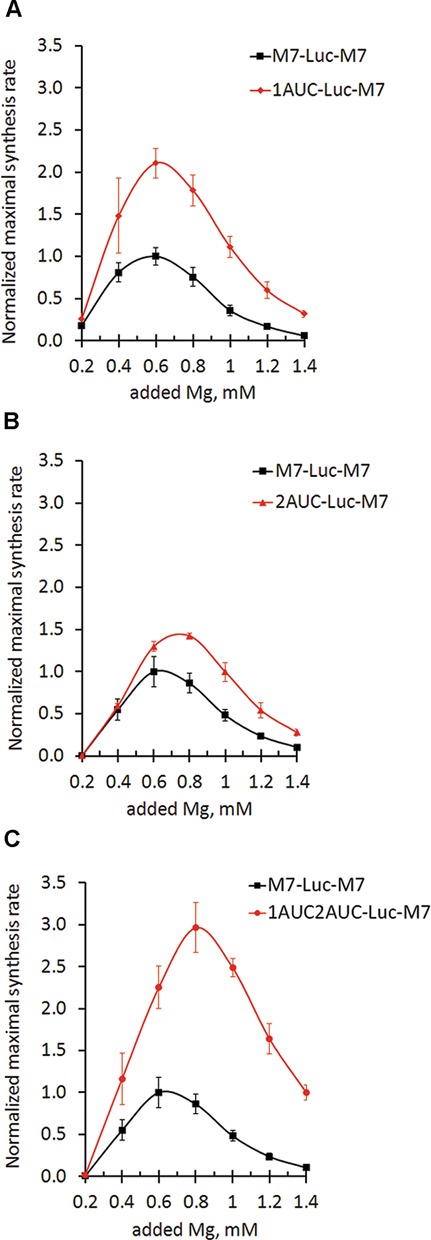
TRPM7 uORF1 and uORF2 reveal different roles in translation of the main coding sequence. Different dependences of translation efficiency on magnesium concentrations: squares represent maximal synthesis rates of translation reactions directed by TRPM7 5′-leader. Translation reactions directed by TRPM7 5′-leader mutant forms are indicated by diamonds for 1AUC-Luc-M7 (**A**), triangles for 2AUC-Luc-M7 (**B**) and circles for 1AUC2AUC-Luc-M7 (**C**). Maximal synthesis rates are normalized to the value of M7-Luc-M7 translation at the optimal magnesium concentration. Error bars represent SD of at least three experiments.

To elucidate the roles of the uORF1 and uORF2 in translation of the main coding sequence, we generated mutant forms of the TRPM7 leader (Figure 2) and tested their abilities to direct translation at different magnesium concentrations (Figure 6). Comparison of the mutant leaders was performed at 60 mM of added potassium acetate as this concentration supported the highest level of translation directed by wild-type TRPM7 5′-leader (Figure 4B).

We found that disruption of the uAUG1 alleviated inhibition of the main coding sequence translation by at least 2-fold (Figure 6A), suggesting an inhibitory role of uORF1 in the translation of the main coding sequence. Disruption of the uAUG2 codon resulted in a shift in magnesium optimum of translation of the main coding sequence (Figure 6B): the mutant 2AUC-Luc-M7 was translated more efficiently at higher Mg^2+^ concentrations than mRNA with wild-type TRPM7 leader. Finally, disruption of both uAUG codons led to an additive effect: increased translation of the reporter sequence with magnesium optimum shifted to higher concentrations (Figure 6C). The observed effects were exclusively due to the effects of magnesium on the efficiency of translation initiation as mRNA quantity was not affected by magnesium concentration in the course of the translation reactions (Supplementary Figure S4).

**Figure 7.**
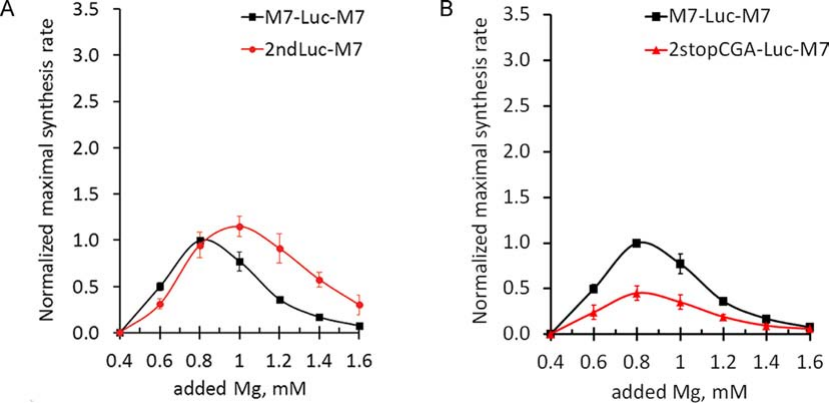
(**A**) TRPM7 uORF2 has higher magnesium optimum of translation initiation. Graph shows dependences of translation rates of uORF2 (circles) and the main coding sequence (squares) on magnesium concentration. (**B**) Stop codon of uORF2 contributes to the initiation of translation of the main reading frame. Graph shows translation rates of 2stopCGA-Luc-M7 (triangles) and M7-Luc-M7 (squares) mRNAs. Maximal synthesis rates were normalized to the value of M7-Luc-M7 translation at the optimal magnesium concentration. Error bars represent SD of at least three experiments.

Since removal of the second uAUG altered the magnesium optimum of translation of the main reading frame, we hypothesized that at increased Mg^2+^ levels translation of the uORF2 may impede ribosomes from proceeding to the main start codon. Therefore, we sought to determine the magnesium dependence of translation of uORF2. To detect translation of uORF2 we fused the luciferase coding sequence to the start codon of the uORF2, leaving its first coding nucleotide triplet intact so as not to disrupt the highly conserved immediate context of the AUG codon (Figure 2, construct 2ndLuc-M7). We found that the uORF2 was indeed translated, and that it was translated more efficiently at higher Mg^2+^ concentrations (Figure 7A).

One of the highly evolutionarily conserved areas within the TRPM7 5′-leader is the region between the stop codon of the uORF2 and the start codon of the main coding sequence (Figure 1C). This region is 5 nucleotides long and is almost identical in all vertebrates. Conservation of the sequence and the precise positioning of the uORF2 stop codon with regard to the main reading frame suggest that this region might play an important role in the regulation of *trpm7* gene expression. We generated a mutant TRPM7 5′-leader where the stop codon of the uORF2 was disrupted by a point mutation (TGA to CGA), extending the uORF2 by 89 nucleotides beyond the main start codon (Figure 2, construct 2stopCGA-Luc-M7). This point mutation caused a 2-fold decrease in translation efficiency of the luciferase reporter without a shift in magnesium optimum (Figure 7B), suggesting that termination of translation of the uORF2 contributes to the initiation at the main start codon but not to its regulation by magnesium.

## DISCUSSION

**Figure 8.**
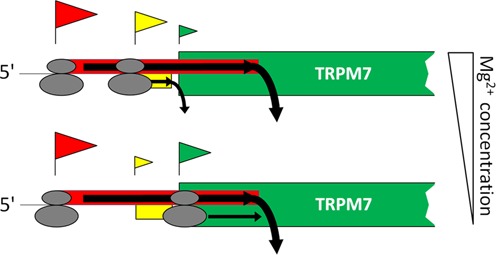
Proposed model of regulation of TRPM7 protein synthesis based on scanning mechanism of translation initiation. Flag above an AUG codon represents an initiation event on that AUG. The size of the flag reflects probability of initiation on the corresponding AUG codon. Most of the time translation is initiated at the first uAUG codon so that the uORF1, which overlaps the start codon of TRPM7 protein, gets translated, preventing synthesis of TRPM7 protein. In a small number of cases 40S scanning subunits are able to bypass the uAUG1 and continue scanning. Under conditions of high magnesium, scanning ribosomal subunits are more likely to initiate at the uAUG2 (upper scenario) and translate the uORF2, reducing the amount of ribosomes reaching the main start codon. Under conditions of low magnesium, scanning 40S subunits are more likely to bypass the uAUG2 and to reach the start codon of TRPM7 coding sequence (lower scenario).

In this study we demonstrate that expression of mammalian *trpm7* gene, encoding for a ubiquitous and essential magnesium ion channel, is regulated by Mg^2+^ concentrations at the level of translation. Furthermore, we elucidate the basic mechanism of this regulation and show that it is achieved via two upstream ORFs within the TRPM7 5′-leader. The uORF1 functions to inhibit overall translation of the main coding sequence, whereas the uORF2 confers specific Mg^2+^ sensitivity of translation directed by TRPM7-leader providing its upregulation at decreased Mg^2+^ concentrations. Based on our findings, we propose a model for the control of *trpm7* gene expression at the stage of translation initiation (Figure 8). Our model incorporates the Kozak scanning mechanism of initiation and the concept of leaky scanning under low Mg^2+^ levels (15,23).

The classical scanning mechanism postulates that the presence of an uORF that overlaps the main ORF is inhibitory for the translation of the main ORF. Our data confirm the inhibitory role of the uORF1 for the translation of the main coding sequence, consistent with the model where the uORF engages a substantial fraction of scanning 40S ribosomal subunits from the translation cycle, making them unavailable for the synthesis of the main ORF product (Figure 8). Moreover, the presence of uAUG codons in similar contexts as the uAUG1 (A^−3^+A^+4^) within 5′-leader regions was shown to significantly reduce translation of downstream reporter sequences (e.g. in the most comprehensive study (24) it inhibited translation of the downstream reporter sequence by 70%). Notably, our results are also in accordance with recent data on ribosome profiling in both mouse and human cultured cells (25,26), demonstrating that the ribosomes initiate at the uAUG1 of TRPM7 with high frequency (Supplementary Figure S5).

The 40S ribosomal subunits that do bypass the uAUG1 encounter the uAUG2 codon. Our data suggest that it is the uAUG2 that adjusts the magnesium optimum of translation of the main coding sequence. We demonstrated that the uORF2 was translated more efficiently under higher Mg^2+^ concentrations (Figure 7). This result is in agreement with both classical (23) and more recent studies (27) showing that the suboptimal context of the uAUG2 (U^−3^+G^+4^) provides initiation of translation more efficiently under conditions of high Mg^2+^ than at decreased Mg^2+^ levels. Thus, under higher Mg^2+^ concentrations the 40S ribosomal subunits that bypass the uAUG1 most probably initiate at the uAUG2, translate the uORF2 and terminate before they reach the main coding sequence (Figure 8, upper scenario). If the Mg^2+^ concentration is low, the uAUG2 is more likely to be bypassed, and 40S ribosomal subunit continues to scan along the mRNA and reaches the main start codon, leading to the synthesis of TRPM7 protein (Figure 8, lower scenario).

TRPM7 5′-leader represents a robust combination of uORFs that governs TRPM7 synthesis according to Mg^2+^ concentration regardless of monovalent salt concentration (Figure 4B). It has long been known that potassium ions compete with magnesium ions for binding sites at the surface of ribosomal subunits (28): the more potassium is bound to the interface of the 40S ribosomal subunit, the more magnesium is needed to occupy specific binding sites and achieve proper initiation-competent conformation. Dynamics of translation reactions directed by β-globin 5′-leader clearly shows that magnesium and potassium ions are able to complement each other (Figure 4A). In contrast, magnesium optimum of translation initiation on TRPM7 5′-leader is highly stable and is not perturbed by varying potassium levels, clearly suggesting that TRPM7 5′-leader utilized a specific and distinct mechanism to “sense” magnesium concentration.

The scanning mechanism of initiation implies one more possible route that can, in principle, lead to the synthesis of TRPM7 protein—a reinitiation event after termination of the uORF2 translation. If reinitiation does happen to some extent, then disruption of the stop codon should lead to a decrease in translation frequency of the main coding sequence. Indeed, we found that the mutant TRPM7 5′-leader with the disrupted stop codon of the uORF2 directed translation 2-fold less efficiently than wild-type TRPM7 5′-leader (Figure 7B). Although the reinitiation is the simplest explanation of our result, alternative scenarios are also possible. For example, the observed inhibitory effect could be caused by spatial interference of moving scanning 40S subunits, elongating ribosomes and positioning of terminating complexes in the area of interest. Further studies are needed to investigate the role of this highly conserved region in the regulation of *trpm7* expression.

On the basis of our results and the current knowledge in the field, we propose the following hypothesis of TRPM7 function and regulation *in vivo*. In a resting cell 95% of cellular magnesium is present in bound form, constituting an essential component of ribonucleic complexes, protein and membranes, whereas only a small fraction (0.5–1 mM) is present in free form (29). Before cell division, all cellular components have to be doubled, requiring a large influx of Mg^2+^. During the G1 phase, as new cellular components are being synthesized, they bind all available free intracellular Mg^2+^, causing a decrease in its concentration. Previous studies have shown that TRPM7 channel activity is regulated by intracellular magnesium: a decrease in Mg^2+^/Mg•ATP activates influx currents through TRPM7. Furthermore, it was shown that TRPM7 currents are specifically increased during the G1 phase of the cell cycle (30). Our study demonstrates that magnesium also regulates *trpm7* gene expression at the level of translation initiation. Specifically, our data suggest that TRPM7 5′-leader allows gradual upregulation of TRPM7 channel synthesis under conditions of decreasing magnesium levels. For example, as seen on Figure 6C, a decrease in magnesium concentration from 1.4 mM to 0.6 mM leads only to 2-fold increase in efficiency of translation directed by a leader without uAUG codons (1AUC2AUC) and to 10-fold increase in efficiency of translation directed by the TRPM7 5′-leader. Thus, TRPM7 protein synthesis is specifically fine-tuned to respond to conditions of low magnesium to promote expression of *trpm7* gene when activity of the channel is most required. It was previously shown that combination of high transcript levels with tight translational control is utilized by living organisms to minimize transcriptional noise, precisely adjusting the amount of synthesized protein to cellular needs in a changing environment (31). The need for such finely tuned regulation is especially important for proteins that display some toxicity when overproduced by the cell. Similar to many other divalent cation channels, TRPM7 is permeable not only to Mg^2+^ but also to other divalent cations, which are potentially toxic, such as Zn^2+^, Ni^2+^, Ba^2+^ and Co^2+^ (32). This promiscuity may explain the high evolutionary conservation of TRPM7 5′-leader organization as it provides a mechanism of tight translational regulation of *trpm7* gene expression.

The sensitivity of magnesium transporter/channel synthesis to intracellular Mg^2+^concentrations could be a universal phenomenon. It is common in prokaryotes and involves sensing of Mg^2+^ via riboswitch structures within 5′-leader sequences of magnesium transporter mRNAs (20,33,34). These sequences control transcriptional elongation by adopting distinct conformations in response to different Mg^2+^ concentrations. Conformations favored under low Mg^2+^ level permit transcription elongation and further magnesium transporter synthesis, whereas conformations adopted at high magnesium levels cause termination of transcription without production of the magnesium transporter. Such a mechanism cannot occur in eukaryotes because (i) transcription and translation are not coupled in eukaryotic cells and (ii) dynamics of translation initiation are highly dissimilar between eukaryotes and prokaryotes. Studies of mechanisms of regulation of magnesium transporter synthesis in eukaryotes so far have been limited to ALR1, a major magnesium transporter in yeast. It was shown that transcript of the *ALR1* gene was a subject for regulated degradation by nonsense-mediated decay pathway due to the presence of a short uORF in its 5′-leader (35). However, data on whether degradation of *ALR1* transcript occurs in response to Mg^2+^ levels remain controversial (36,37). In this study we found the first example of a magnesium channel synthesis being controlled by Mg^2+^ concentration in a vertebrate at the stage of translation initiation. It relies on the fact that in eukaryotes the 40S ribosomal subunit binds 5′-cap structure and starts the unidirectional mRNA scanning in search of an AUG codon. Our data demonstrate that magnesium sensitivity of TRPM7 protein synthesis is based on the differential probability of uAUG recognition during this scanning process. Since the TRPM7 5′-leader uORFs are conserved among all vertebrates, it is likely that their function in regulating TRPM7 translation is likewise conserved. Future studies will elucidate the finer details of the mechanism of this regulation and the extent of its evolutionary conservation.

## SUPPLEMENTARY DATA

Supplementary Data are available at NAR Online.

SUPPLEMENTARY DATA
