# Supplementary material for: Identification of a Mg2+-sensitive ORF in the 5′-leader of TRPM7 magnesium channel mRNA
Source: Nucleic Acids Res. 2014 Oct 17;42(20):12779–88. doi: 10.1093/nar/gku951 (PMC4227784; doi:10.1093/nar/gku951)
Supplement: SUPPLEMENTARY DATA [file supp_42_20_12779__index.html]

Identification of a Mg2+-sensitive ORF in the 5′-leader of TRPM7 magnesium channel mRNA — Identification of a Mg2+-sensitive ORF in the 5′-leader of TRPM7 magnesium channel mRNA — SUPPLEMENTARY DATA 

# Identification of a Mg2+-sensitive ORF in the 5′-leader of TRPM7 magnesium channel mRNA

## SUPPLEMENTARY DATA

**Files in this Data Supplement:**

- SUPPLEMENTARY DATA
