# Supplementary material for: Identification of a Mg2+-sensitive ORF in the 5′-leader of TRPM7 magnesium channel mRNA
Source: Nucleic Acids Res. 2014 Oct 17;42(20):12779–88. doi: 10.1093/nar/gku951 (PMC4227784; doi:10.1093/nar/gku951)
Supplement: SUPPLEMENTARY DATA [file supp_gku951_nar-01575-x-2014-File010.pdf]

Supplemental figures

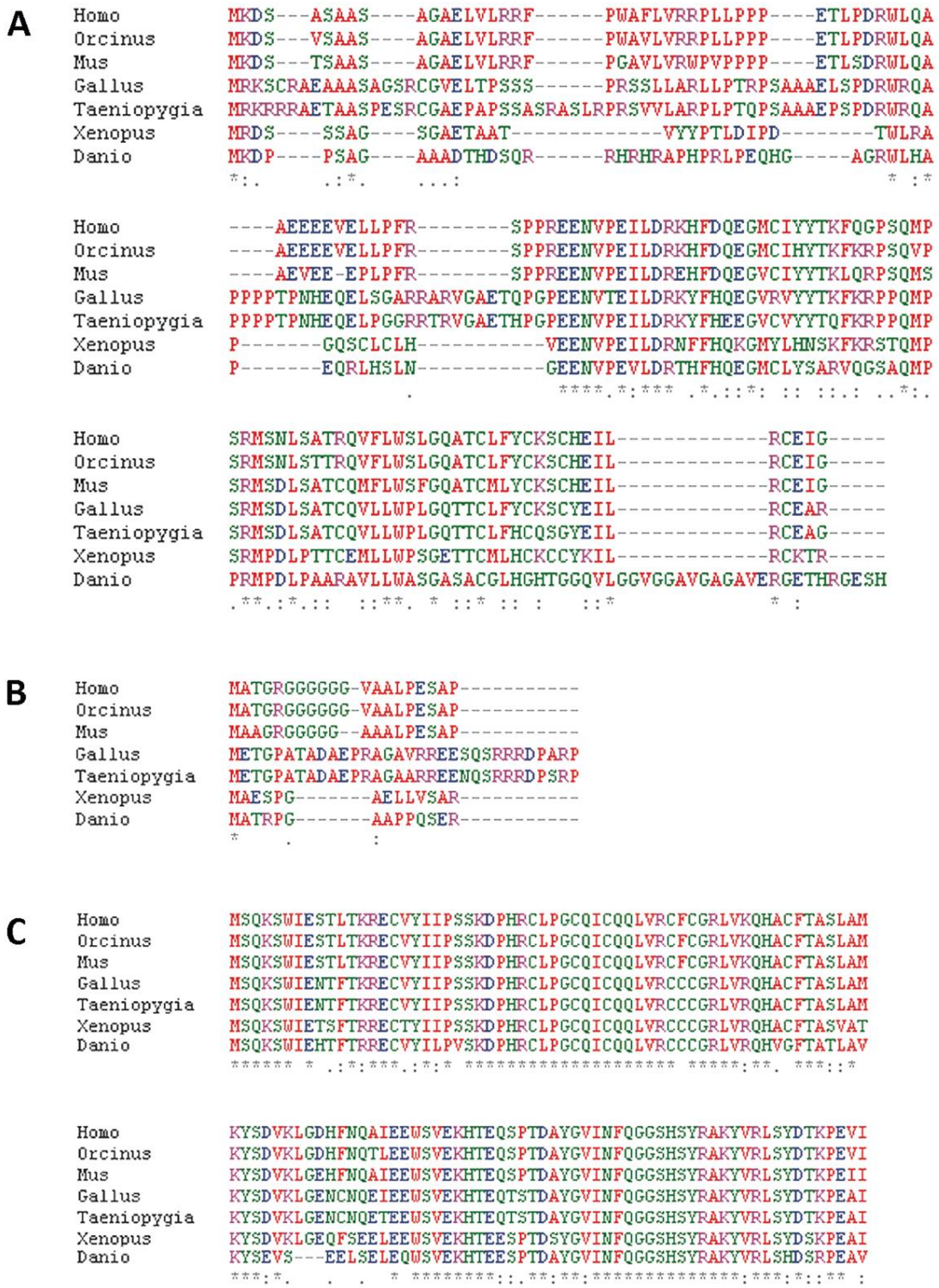

Figure S1.
Peptide sequences encoded by the uORFs of TRPM7 mRNA are not evolutionarily conserved.
(**A**) Peptides encoded by the uORF1, (**B**) peptides encoded by the uORF2 and (**C**) N-termini of proteins encoded by the main reading frame of TRPM7 mRNA. The alignment was performed using ClustalOmega software [38]. The uORF1 overlaps the main reading frame; a black arrow indicates the beginning of the main coding sequence in +1 frame relative to the frame of the uORF1.

A B

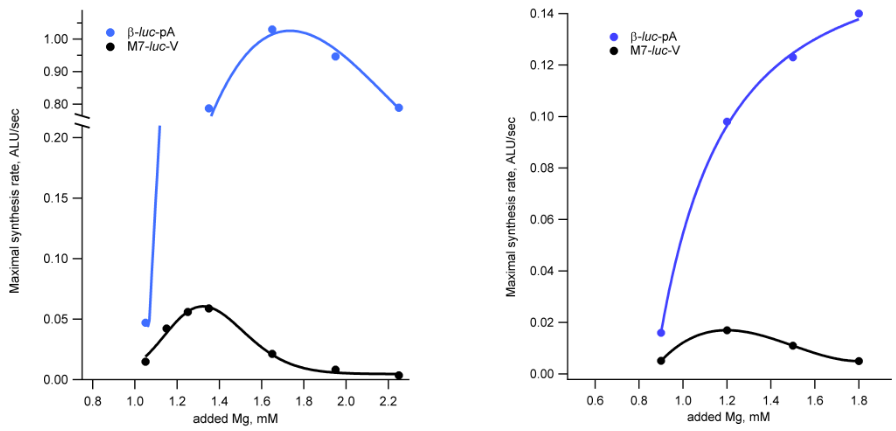

Figure S2

Translation directed by TRPM7 5'-leader is inefficient and can be activated by lowering magnesium concentration.

Dependence of maximum synthesis rates on magnesium concentration for translation reactions directed by β-globin leader (blue circles) and TRPM7 5’-leader (black circles) in a wheat germ (**A**) and Krebs2 (**B**) systems.

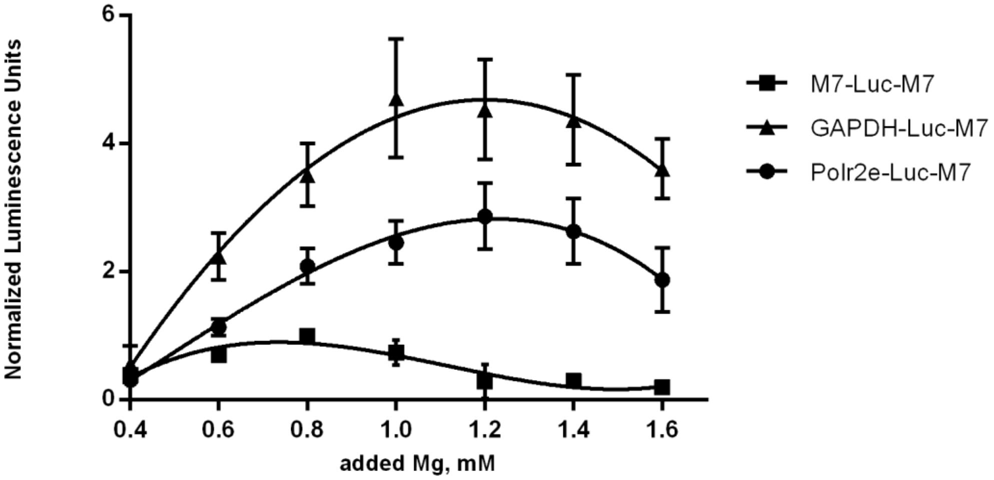

Figure S3

Translation directed by TRPM7 5'-leader has lower magnesium optimum than translation directed by other cellular 5'-leaders.

Dependence of maximum synthesis rates on magnesium concentration for translation reactions directed by TRPM7 (squares), GAPDH (triangles) and Polr2e (circles) 5'-leaders in the HEK293 system. Translation reactions were performed at 120 mM of added potassium acetate.

Construct	B-Luc-M7			M7-Luc-M7			1AUC-Luc-M7			2AUC-Luc-M7			1AUC2AUC-Luc-M7		
Added Mg, mM	1.0	0.6	1.0	1.0	0.6	1.0	1.0	0.6	1.0	1.0	0.6	1.0	1.0	0.6	1.0
Time, min	0	40	40	0	40	40	0	40	40	0	40	40	0	40	40

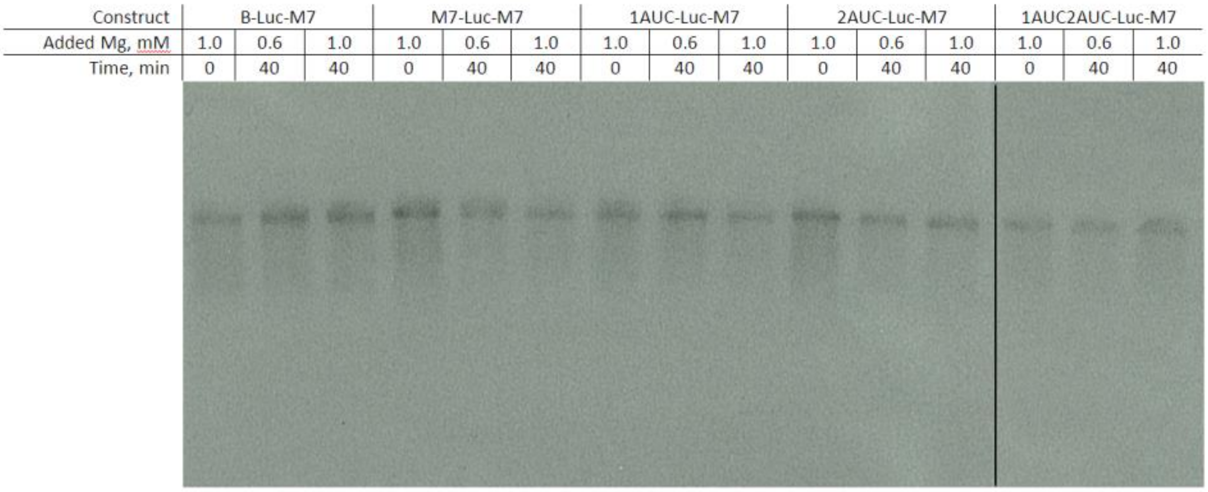

Figure S4
RNA levels in *in vitro* translation reactions are not affected specifically by varying magnesium concentrations and are stable during the course of the reaction.
Northern blotting analysis of total RNA extracted from translation reactions conducted at the indicated concentrations of added magnesium at the indicated time points. A radioactive probe corresponding to the luciferase coding region was used to visualize quantity of the exogenous mRNA in the translation reactions. A composite picture is presented. Black line represents the border between two parts of the same film that were stacked together.

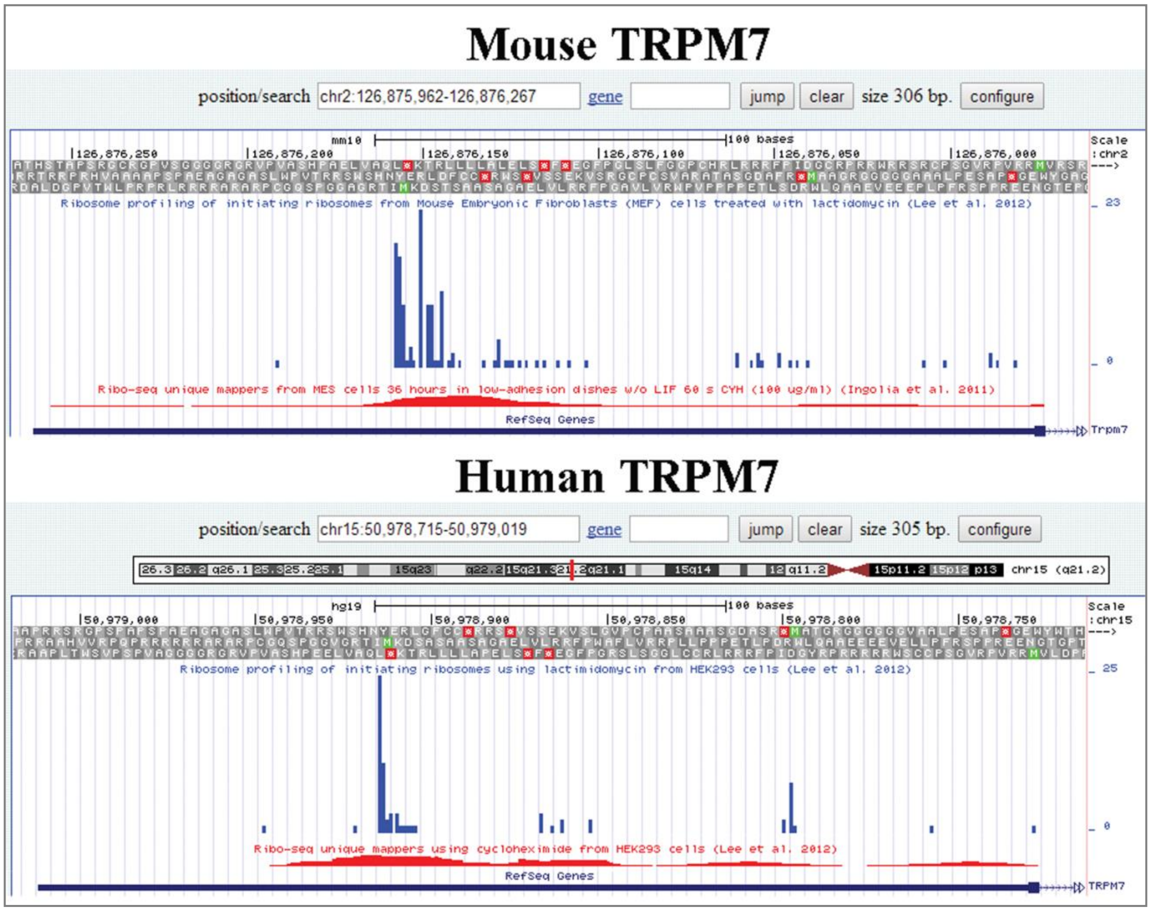

Figure S5

TRPM7 uAUG1 codon is used with high frequency both in human and mouse cultured cells. Distribution of initiating ribosomes along TRPM7 5'-leader (obtained using lactimidomycin inhibitor) is shown in blue bars; distribution of elongating and initiating ribosomes (obtained using cycloheximide inhibitor) is shown in red bars. Amino acid sequences for all 3 coding frames are shown in grey at the top of each panel (three green squares represent methionine residues encoded by the two uAUG and the start codon of TRPM7 mRNA; red squares indicate stop codons). The maps of ribosome footprint densities were obtained by GWIPS-viz genome browser [39], http://gwips.ucc.ie/, on the basis of several ribosome profiling studies [25,26].

Supplemental Methods

Description of the constructs tested in WGE and Krebs2 translation systems
β-Luc-pA construct is described in [14]. M7-Luc-V construct contained murine TRPM7 5'UTR followed by sequence encoding for firefly luciferase and 3'UTR region from pGL4.50 vector (Promega).

***In vitro* translation in WGE and Krebs2**
Wheat germ extract (WGE) was prepared from germs of wheat, sort Kazakhstanskaya 4, essentially according to the protocol in [40] with a modification in the germ washing procedure described in [41].The concentration of the obtained WGE was 246 OD_{260}/ml. The final translation mixture contained 20% v/v WGE, 100 µg/ml creatine phosphokinase, 500 U/ml RNase inhibitor, 50 mg/ml yeast total tRNA, 0.1 mM each amino acid, 1 mM ATP, 0.6 mM GTP, and 16 mM creatine phosphate in 20 mM HEPES-KOH buffer pH 7.6 with 1.25 mM $Mg(OAc)_2$, 2.5 mM DTT, 0.25 mM spermidine and 0.1 mM luciferin. The total K^+ concentration in the translation system was 35 mM.
Krebs-2 cell extracts were prepared from mouse Krebs-2 ascites cells as described in [22]. The final translation mixture contained 50% v/v Krebs-2 extract, 100 µg/ml creatine phosphokinase, 500 U/ml RNase inhibitor, 50 mg/ml calf total tRNA, 25 µM each amino acid, 1 mM ATP, 0.2 mM GTP, and 8 mM creatine phosphate in 20 mM HEPES-KOH buffer pH 7.6 with 0.6 mM $Mg(OAc)_2$, 100 mM KOAc, 1 mM DTT, 0.5 mM spermidine and 0.1 mM luciferin.
Reaction components were mixed on ice, adjusted to 80% of the final volume, and incubated for 2 min at 25°C (WGE) or 30°C (Krebs-2). 2 µl of preheated 5-fold concentrated mRNA/Mg^{2+} mixture were diluted with 8 µl of the prepared reaction mixture and immediately put into the temperature-controlled cell of a Chemilum-12 multichannel luminometer. The streaming data of light emission were collected on the computer as a kinetic curve.

Northern hybridization analysis of RNA stability during *in vitro* translation
Total RNA was isolated from *in vitro* translation reactions at the indicated time points using RNeasy kit (Qiagen). 250 ng of the extracted RNA was separated by electrophoresis in a formaldehyde-containing 1.2% agarose gel and transferred onto a nylon membrane. A probe used for hybridization was prepared by PCR amplification of a 300 bp DNA fragment coding for a C-terminus of the firefly luciferase. The amplified double-stranded fragment was gel-purified using Qiagen Gel Purification Kit, dephosphorylated using alkaline phosphatase (Roche) and radioactively labeled by T4 polynucleotide kinase (New England Biolabs) in the presence of [γ-^{32}P]-ATP (Perkin Elmer).

Sequences of 5'- and 3'-leaders used in this study

Human β-globin leader:
5'-GGTACCACATTTGCTTCTGACACAACTGTGTTCACTAGCAACCTCAAACAGACACC**ATG**

Murine GAPDH leader:
5'-GGTACCTCTCTGCTCCTCCCTGTTCCAGAGACGGCCGCATCTTCTTGTGCAGTGCCAGCCTCGTCCCGTAGACAAA**ATG**

Murine Polr2E leader:
5'- GGTACCCAGGCTCCGTGCTGGCAGCCACGAGCGCGCGTGCACAGTTGTTTGGGAAGCGCGACAGC**ATG**

M7-Luc-M7 construct:
GGTACCCGACGGCCCCGTCACGTGGCTGCCGCGGCCCCGTCTCCGGCGGAGGCGGGCGCGGGCGCGTCCCTGTGGCCAGTCA
CCCGGCGGAGCTGGTCGCACAATTATGAAAGACTCGACTTCTGCTGCTAGCGCTGGAGCTGAGTTAGTTCTGAGAAGGTTTCC
CGGGGCTGTCCTTGTTCGGTGGCCCGTGCCACCGCCTCCGGAGACGCTTTCCGATAGATGGCTGCAGGCCGCGGAGGTGGAG
GAGGAGCCGCTGCCCTTCCGGAGTCCGCCCCGTGAGGAGA**ATG** -Luciferase coding sequence from pGL4.50-**TAA**
TGCTGAGTCATTGGTTTTTGCCTACACTTCACAAAAGTGTAACTGTCAGTTTTCCTTTCGGGGGAATTGATGATATAGGAAGAT
GTGTGCAAAATGAGCTTGCTGGCCCCACACATAGTCTAGAGGTAATGTTCTCATTGAAAAACGCCTGGAGGCTGCAGATGACA
GCTGGAAAGTGCTAGCTGGCAGAGAGTCAGTGCTCTCGGCTGGTGAAGGGCGGGAACCTTGCTGCTGAGAGTGGTGGTTCTC
TCACCTGGTGCAGGACCATTAACCAAAGTCAAGTCTTCAGATTTGATTGGCTGCTCAGTCACAGCCATTCAGCTAAGGAAACTA
AATTGCGCAGCTTTTTAAATGGCTGAAGTCTTCCTCAGTTTGTGCTCTATGATAATGATGTTAGCTCTCAACTAGGTGTTTGTGG
CCACGGGAGAACTACTCCTTACAATTTTGCTTCACAGGCATGTTACAAAGCCTGCACTGAAAACCGTTTGTCTTCCCTCTCTCCC
TCCCTCTTTTCCCTGTAGTATTGAGGATCAAACCCAGGGCCTCATGAAGACCATTTTCTAAGAGACATTTTATTTAAGAATCAAC
TATAGAGTCTATGTTTATGGATACAGCCAGTTTTTGTTAAACAAAACCTGAATTGTGCAAAAGGGTTTTTTAACATTTATCAATG
TTAAGTAAAAGAAAGCCATGATAAATAAGAATTAACTCACTGTTCAATGGGTGTTTCCTGTGAGGAAGGTTACAGTTGTAACA
GCCTGCAGTTGCATACATCTCCAAAGATTTACAGACTTAGTGTATCAAATCAGAGTGTCATGTGAGCTCTCACATTGAAAATTCT
ATAGGAATGTGTCAATGTGAATTCTATTTCTGGTACTTAAGAAATCAGTTGTTGGATTATCCTTATACAGTATAGGGAGATCAC
AATACAACTTTATGCCAATAAAATCTAACTTAATTGCCCAGATATTTTTGCATATTTAGCAACAAGAAAAGCTTATCATTTGACT
CAAGTTTTATGCTTTCTCTTTCTTTTCATTTCCTAGGTACTAATTTTAATTTTTATTTGGAAGGAGCAGTGTAAAGCTTACTTGTAT
TCAATAGTGTATCTCATAGATACAGACAAGGCCGCAGAGATAAGCTGTTAAATAGTGTTTAATGTTGATGTGGAGAGAAAGGT
GTATTACTTAAAAATACTATACCATATACGTTTTGTATATCATTAAATCTTTAAAAGAAATTAAATTTATTCTTGTTTACAAAAAA
AAAAAAAAAAAAAAAAAAAAAAAAAAAAGGGCGGCCGCTCGAGTCTAGAGGGCCCGTTTAAAGGATCC

Restriction sites (KpnI on the 5'-end and BamHI on the 3'-end) that were used for insertion of the sequence into pcDNA3.1 vector are underlined. The uAUG1 is highlighted with red. The uAUG2 and the stop codon of the uORF2 are highlighted with yellow. Luciferase coding sequence is marked by green and followed by murine TRPM7 3'UTR.

Supplemental references

38. Sievers F, Wilm A, Dineen D, Gibson TJ, Karplus K, Li W, Lopez R, McWilliam H, Remmert M, Söding J, et al. (2011) Fast, scalable generation of high-quality protein multiple sequence alignments using Clustal Omega. *Molecular systems biology* **7**: 539.
39. Michel AM, Fox G, Kiran AM, De Bo C, O'Connor PBF, Heaphy SM, Mullan JPA, Donohue CA, Higgins DG, Baranov PV (2014) GWIPS-viz: development of a ribo-seq genome browser. *Nucleic acids research* **42**: D859–64.
40. Erickson AH, Blobel G (1983) Cell-free translation of messenger RNA in a wheat germ system. *Methods in enzymology* **96**: 38–50.
41. Shirokov VA, Kommer A, Kolb VA, Spirin AS (2007) Continuous-exchange protein-synthesizing systems. *Methods in molecular biology (Clifton, NJ)* **375**: 19–55.
